# Gastrothorax or tension pneumothorax: A diagnostic dilemma

**DOI:** 10.4103/0974-2700.76821

**Published:** 2011

**Authors:** Sarvesh P Singh, Subin Sukesan, Usha Kiran, Neeti Makhija

**Affiliations:** Department of Cardiac Anaesthesia, Cardiothoracic Centre, All India Institute of Medical Sciences, New Delhi 110029, India

**Keywords:** Gastrothorax, tension, pneumothorax, diagnostic dilemma

## Abstract

Gastrothorax, a rare complication following thoracoabdominal aortic aneurysm repair, is reported. The clinical features of a gastrothorax and tension pneumothorax are similar and thus, a gastrothorax can masquerade as a tension pneumothorax. The diagnosis is made by a high level of clinical suspicion, chest X-ray shows a distended stomach with air fluid levels and a computerised tomography is useful in assessing the diaphragm and establishing the positions of the various intra-abdominal organs. Also, the risk of an intercostal drainage tube placement and the role of nasogastric tube in avoiding the development of a tension gastrothorax is highlighted.

## INTRODUCTION

Thoracoabdominal aortic aneurysm (TAAA) repair has been associated with a number of complications such as postoperative hemorrhage, atelectasis, pneumonia, acute respiratory distress syndrome (ARDS), tension pneumothorax and ischemia-induced injury to the abdominal viscera and spinal cord.[1]

We, hereby, report the case of a 35-year-old hypertensive female who underwent a TAAA repair with a collagen-impregnated polyester vascular graft (Cardial, C. R. Bard, France). Postoperatively, she was admitted in the intensive care unit (ICU) and was extubated on postoperative day 1 with normal hemodynamic parameters. On day 6, she complained of epigastric burning sensation with retching, which was relieved with a combination of ranitidine and pantoprazole. Later in the day, these symptoms became refractory to medication. Subsequently, the patient was kept nil per orally and a nasogastric tube (NGT) was inserted. A workup was initiated for gastroesophageal reflux disease (GERD), acute pancreatitis, subacute intestinal obstruction, paralytic ileus, and mesenteric ischemia.[2] During the night, the symptoms of patient worsened along with tachycardia, tachypnoea, hypotension, and diaphoresis. Clinical examination revealed a tympanic percussion note over left hemithorax with absent breath sounds. Heart sounds and normal breath sounds were heard to the right of the sternum.

**Figure 1:**
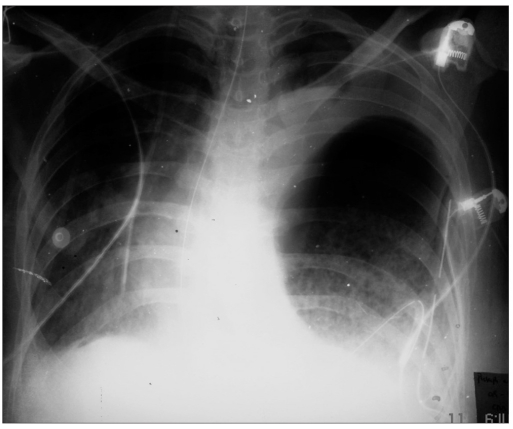
Chest X-ray showing a distended stomach in left hemithorax and contralateral mediastinal shift. Left lung is compressed

**Figure 2:**
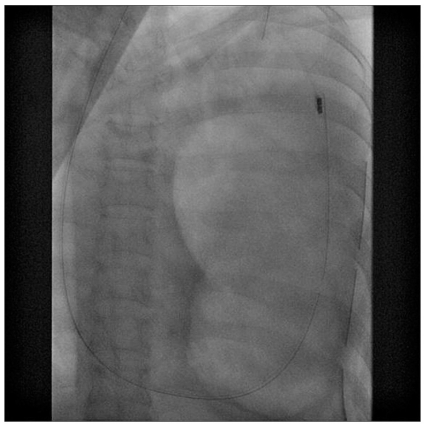
Fluoroscopy image revealing the tip of nasogastric tube in the left hemithorax

**Figure 3:**
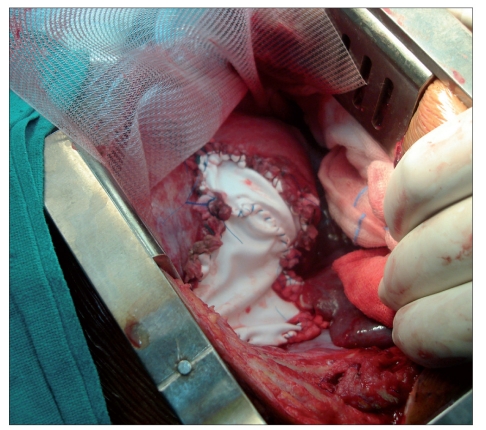
Surgical repair of diaphragm using a polytetrafluoroethylene patch (Gortex patch) and prolene mesh

A provisional diagnosis of left tension pneumothorax was made, and an intercostal drainage (ICD) tube was placed immediately. The symptoms were not relieved much after ICD placement. A follow-up chest radiograph [Figure 1] revealed a diaphragmatic rupture with a distended stomach in left hemithorax; shifting the mediastinum to the contralateral side and compressing the lung. Thereafter, a fluoroscopic confirmation was done which unveiled the tip of NGT to be in the left hemithorax [Figure 2]. An emergency surgical repair of diaphragm with a polytetrafluoroethylene (PTFE) patch and prolene mesh was performed [Figure 3]. There was marked clinical improvement in clinical status of patient after the surgery. She was discharged on 18^th^ postoperative day.

Gastrothorax in adults usually develops after traumatic rupture of the diaphragm and can be misdiagnosed as tension pneumothorax.[3] In this case, the clinical examination misled the attending surgeon and an ICD was placed with tension pneumothorax as diagnosis. Interestingly, this is one of the few cases in which placement of an ICD could have led to a catastrophe in the form of perforation of stomach. Because the conventional trocar and cannula are not used in our center for ICD placement, this complication was unknowingly avoided. Another complication that could have occurred was a tension gastrothorax.[4] A tension gastrothorax occurs due to trapping of air in the stomach. This may result from air swallowing in respiratory distress or insufflation of air during bag mask ventilation or gastroscopy.[5] This was avoided in the first place because a NGT was already inside the stomach. How did the NGT manage to camouflage is also important in this case? Although, conventionally, checked by the auscultation method; some personnel do rely on the passive flow of gastric contents through the tube. It may be stressed that auscultation in epigastrium not only confirms the position of NGT in stomach, but also implies that stomach is in the abdominal cavity. Therefore, even if there is passive flow of gastric contents from the NGT, auscultation of air gush in the epigastrium is still required. Gastric herniation into the thorax on seventh postoperative day was highly unexpected and therefore misdiagnosed as tension pneumothorax, a more common complication.

## References

[1] Etz CD, Luozzo GD, Bello R, Luehr M, Khan MZ, Bodian CA (2007). Pulmonary complications after descending thoracic and thoracoabdominal aortic aneurysm repair: Predictors, prevention, and treatment. Ann Thorac Surg.

[2] Talley NJ, Phung N, Kalantar JS (2001). Indigestion: When is it functional?. BMJ.

[3] De Jager CP, Trof RJ (2004). Images in clinical medicine: Gastrothorax simulating acute tension pneumothorax. N Engl J Med.

[4] Van Berkel-Mijnsbergen JY, Loosveld OJ, Vos LD (2007). Abdominal pain with unexpected pulmonary consequences: Diagnosis: Tension gastrothorax. Neth J Med.

[5] Mortelmans LJ, Jutten GC, Coene L (2003). Acute post-traumatic tension gastrothorax a tension pneumothorax-like injury. Eur J Emerg Med.

